# Proceedings of the 2024 Transplant AI Symposium

**DOI:** 10.3389/frtra.2024.1399324

**Published:** 2024-08-29

**Authors:** Sara Naimimohasses, Shaf Keshavjee, Bo Wang, Mike Brudno, Aman Sidhu, Mamatha Bhat

**Affiliations:** ^1^ Division of Gastroenterology, Toronto General Hospital, Toronto, ON, Canada; ^2^ Ajmera Transplant Center, University Health Network, Toronto, ON, Canada; ^3^ Department of Innovation, University Health Network, Toronto, ON, Canada; ^4^ Department of Laboratory Medicine and Pathobiology, The Temerty Centre for AI Research and Education in Medicine, Toronto, ON, Canada; ^5^ Department of Computer Science, University of Toronto, Toronto, ON, Canada

**Keywords:** artificial intelligence, machine learning, prognostication models, transplant medicine, clinical deployment

## Abstract

With recent advancements in deep learning (DL) techniques, the use of artificial intelligence (AI) has become increasingly prevalent in all fields. Currently valued at 9.01 billion USD, it is a rapidly growing market, projected to increase by 40% per annum. There has been great interest in how AI could transform the practice of medicine, with the potential to improve all healthcare spheres from workflow management, accessibility, and cost efficiency to enhanced diagnostics with improved prognostic accuracy, allowing the practice of precision medicine. The applicability of AI is particularly promising for transplant medicine, in which it can help navigate the complex interplay of a myriad of variables and improve patient care. However, caution must be exercised when developing DL models, ensuring they are trained with large, reliable, and diverse datasets to minimize bias and increase generalizability. There must be transparency in the methodology and extensive validation of the model, including randomized controlled trials to demonstrate performance and cultivate trust among physicians and patients. Furthermore, there is a need to regulate this rapidly evolving field, with updated policies for the governance of AI-based technologies. Taking this in consideration, we summarize the latest transplant AI developments from the Ajmera Transplant Center’s inaugural symposium.

## 1 Introduction

Since its first description in the 1950s by Alan Turing, there has been huge interest in the applicability of artificial intelligence (AI) in clinical practice, particularly given its potential to analyze large datasets (1). However, progress in the medical field had remained limited until the early 2000s, when the development of deep learning (DL) methods, which involved the creation of artificial neural networks, extended the capability of AI for data analysis beyond a fixed model of pattern recognition inputted by a human operator, to developing its own representations in a learning algorithm, recognizing new patterns among vast quantities of unstructured data (1–3). This sparked a wave of studies exploring the utility of DL in the identification of novel patterns, which could assist in improving diagnostic techniques and the prediction of therapeutic responses. In 2017, *Arterys* was the first healthcare cloud-based DL application to be approved by the US Food and Drug Administration (FDA). *Arterys* uses cardiac magnetic resonance imaging (MRI) exams to estimate ventricular function (4). To date, there have been 694 approved AI-based medical applications (5). It is a rapidly growing market, reaching a value of 9.01 billion USD in 2023, with a forecasted compound annual growth rate of 40% (6).

In line with these developments, there has been an array of research exploring the versatility of AI in different areas of healthcare. Within the clinical setting, DL models have demonstrated utility in aiding clinical decision-making, processing and combining multi-omics profiles with clinical, demographic, and epidemiologic data and diagnostic results, thereby enabling the practice of precision medicine, in which treatment is tailored to the specific needs of the individual, rather than being confined to standardized “one-size fits all” protocols (7–9). In addition, AI-based tools have been used to improve diagnostic testing, assisting with medical imaging and histopathology review, which has improved patient monitoring methods, engagement, and compliance (10, 11). The prospective benefits have been shown to extend to all aspects of healthcare, including medical education, research, development, and even healthcare organization, in which it has been shown to improve workflow and resource utilization, reducing costs and medical errors (7, 12, 13).

However, despite the potential for revolutionizing healthcare, the successful deployment and implementation of DL models in day-to-day practice is not without its challenges. Essentially, the models must be derived using robust datasets obtained from the target population, be well calibrated, and undergo extensive validation. This is critical as erroneous assumptions can adversely impact management and lead to harm. One notable example was observed with the external validation of the Epic Sepsis Prediction Model in the US (14). Furthermore, there are few randomized clinical trials and prospective studies in DL, and peer-reviewed publications are not a prerequisite for FDA approval (15). Assessing the reproducibility of DL research is also challenging due to limitations in the availability and accessibility of the datasets and codes from which the models are generated (15). In addition, AI-based models are generated within the confines of existing published data, which have significant age, gender, and racial biases (16–18). This inability to extrapolate beyond the inputted data comes with a significant risk of amplifying existing biases and disparities in healthcare (15–18).

Transplant medicine is a nuanced specialist area, with many variables at play. At present, there is room for significant improvements in our delivery of care, including prioritization fairness in the organ allocation process, optimizing donor-recipient matching, improving graft survival, and practicing precision medicine by tailoring immunosuppression levels to reduce the risk of rejection while addressing metabolic risk factors to improve long-term outcomes.

In this position paper, we detail the proceedings from the Ajmera Transplant Center’s inaugural Transplant AI Symposium, summarizing the latest advancements in the use of AI-based tools, current challenges, barriers to implementation, ethical considerations, and future directions aimed at modernizing and improving care for transplant recipients.

## 2 Symposium highlights

### 2.1 The future of AI in transplant

The inaugural symposium was opened by the keynote speaker, Dr. Alexandre Loupy, from the Necker Hospital in Paris. Introducing a multimodal approach to the use of AI in transplantation, he detailed his stepwise approach from the research and development of DL models to extensively validated decision support systems that can be successfully incorporated in day-to-day clinical practice.

Dr. Loupy highlighted the three main pillars of his AI journey. The first was centered around the development of the multimodal prognostication system for allograft loss in renal transplant recipients (*iBox*) in 2012, using a prospectively derived comprehensively phenotyped cohort of renal transplant recipients (19–21). The prognostication system incorporates data that are easily accessible in all transplant centers, such as donor and patient characteristics, medical history, histopathology, treatment regimens, and longitudinal clinical parameters. Designing the model exclusively for the renal transplant recipient helped increase the specificity of the algorithm, and evaluating it in a diverse patient population improved the generalizability of the model. During development, there was extensive internal validation, including a methodological validation of the statistical model required to generate the prognostic scoring system, guaranteeing a causal DL model, with parameters used for scoring being directly related to independent predictors of long-term allograft failure in renal transplant recipients. This was followed by an external independent validation in multiple transplant centers worldwide, with subsequent involvement in numerous randomized controlled trials (RCTs) demonstrating the translatability and relevance in geographically distinct populations. The model was subsequently involved in numerous RCTs (see Table 1) and has received endorsement from transplant societies and the European Medicines Agency (EMA), and is currently under review with the FDA. The *iBox* system currently involves data from 27,000 patients across 44 centers, in 14 different countries spanning 5 continents. The *iBox* score is now validated as a surrogate of long-term allograft survival and is used as a clinical endpoint in clinical trials. Work is under way to develop an equivalent pediatric model.

**Table 1:** Summary of validation studies and randomized controlled trials involving the *iBox* prognostication system.

Study	Description	Population	Outcome	Results
Prediction system for the risk of allograft loss in patients receiving kidney transplants: International derivation and validation study (20) 2019	Development of an integrative system for predicting long-term kidney allograft failure: ***iBox***, consisting of clinical, histological, functional, and immunological variables with multicenter, international validation. *Further validation in RCTs (BENEFIT^a^, BENEFIT-EXT^b^, CERTITEM^c^ and (Borteject^d^)	• Derivation cohort (*n* = 4,000) prospectively enrolled renal transplant recipients across France (both living and deceased donors) from Jan 2005 to 2014 • Validation cohort (*n* = 3,557), prospectively enrolled recipients across Europe and the US between 2002 and 2014 • Median follow-up of 7.65 years following transplantation	Allograft loss	• A total of *n* = 1,067 (14.1%) allograft failures over a median follow-up of 7.12 years between the derivation and the validation cohorts • Good discriminative performance following internal and external validation **C index:** 0.81 (95% CI 0.78–0.84) in Europe, 0.80 (95% CI 0.76–0.84) in US
Application of the ***iBox*** prognostication system as a surrogate endpoint in the transform^e^ randomized controlled trial (22) 2021	Proof-of-concept study utilizing the ***iBox*** prognostication system as a surrogate marker of long-term outcomes in renal transplant recipients, projecting up to 11 years post randomization, in a large RCT	• *n* = 940 renal transplant recipients treated with everolimus combined with reduced exposure calcineurin inhibitor matched with *n* = 932 recipients treated with mycophenolate mofetil combined with standard exposure calcineurin inhibitor • ***iBox*** prognostication scores calculated at 1-year visit	Long-term allograft survival projections for 4, 6, and 11 years	• First application of a validated risk scoring system for predicting long-term allograft survival in an RCT • First application of the validated ***iBox*** prognostication system as a surrogate endpoint in renal transplant recipients • Results support the use of the model as a clinical trial simulation tool • Everolimus combined with reduced exposure calcineurin inhibitor was found to be non-inferior to mycophenolate mofetil with standard calcineurin inhibitor therapy
Validation of a prediction system for risk of kidney allograft failure in pediatric kidney transplant recipients: an international observational study (23) 2023	Validation of the ***iBox*** kidney allograft risk prediction system in pediatric renal transplant recipients	• *n* = 1,359 pediatric renal transplant recipients across 20 centers in Europe and the United States	Allograft loss	• A total of 706 kidney allograft evaluations performed over a median follow-up time of 9.1 months post-transplant • ***iBox*** demonstrated accurate calibration and good discrimination for predicting outcomes **C index:** 0.81 (95% CI 0.75–0.87)

^a^ BENEFIT, Belatacept evaluation of nephroprotection and efficacy as first-line immunosuppression, ClinicalTrials.gov ID NCT00256750.

^b^ BENEFIT-EXT, Belatacept evaluation of nephroprotection and efficacy as first-line immunosuppression trial-extended criteria donors.

^c^ CERTITEM, Progression of renal interstitial fibrosis/tubular atrophy (IF/TA) according to epithelial-mesenchymal transition (EMT) and immunosuppressive regimen (everolimus based versus CNI based) in *de novo* renal transplant recipients, ClinicalTrials.gov ID NCT01079143.

^d^ BORTEJECT, Bortezomib in late antibody-mediated kidney transplant rejection, ClinicalTrials.gov ID NCT01873157.

^e^ TRANSFORM, Advancing renal transplant efficacy and safety outcomes with an everolimus-based regimen, ClinicalTrials.gov ID NCT03474003.

The second pillar builds on the first, incorporating data on HLA antibodies with novel biomarkers of graft pathology, including immunological markers (21, 24–28) and donor-derived cell free (DD-CF) DNA (24, 26, 27, 29–32), in addition to novel molecular technologies (27, 33–38), which enable more detailed allograft phenotyping in a multimodal algorithm-based decision support system (32, 39–43). There is currently an ongoing multicenter prospective RCT (the EU-TRACER IMPACT study) involving eight European transplant referral centers assessing whether the integration of these additional models, including DD-CF DNA, can further increase the model’s accuracy in predicting allograft failure and patient mortality.

Finally, the last pillar focuses on using AI to improve diagnostics and disease classification through precision diagnostic platforms that incorporate digital histological analysis, including digital spatial profiling and single-cell pathology with diagnostic techniques involving the “molecular microscope” system and non-invasive biomarkers, such as DD-CF DNA. This multidimensional approach to diagnostics has already helped amend the Banff diagnostic classification of antibody-mediated rejection (ABMR) in renal transplant recipients (35, 44), and pursuing a multidimensional approach to diagnostics holds further promise, including in improving precision diagnostics for xenotransplant recipients.

Dr. Andrew T. Sage, from the Latner Thoracic Research Laboratories, then discussed the development of the *InsightX* AI model (Transplant Hepatology and Machine Learning Departments, University of Toronto, Toronto, ON, Canada) for assessing organ injury and predicting patient outcomes during lung transplantation. Organ availability remains a significant issue in transplantation, not excepting individuals awaiting a lung transplant, with over 110,000 people in the US on the waiting list, which has a person added to it every 9 min (45). Considerable demand in the face of a supply shortage has a significant impact on waitlist mortality, with 17 people on the lung transplant waiting list dying daily (45). Lung allografts tend to be particularly susceptible to ischemia-reperfusion injury, highlighting the importance of optimizing organ preservation (46). This has led to the use of *ex vivo* lung perfusion (EVLP) to help restore lung physiology, reducing the rates of ischemia-reperfusion injury, and facilitate the assessment of impaired donor lungs (47). Interestingly, the machine also provides a unique platform for obtaining serial physiological, biochemical, and imaging data. Dr. Sage and his team compiled longitudinal measurements recording over 100 variables from donor lungs, including cytokine production, lactate, electrolytes, airway pressures, vascular resistance, and dynamic and static compliance. These high-resolution data profiles were then used to create the first AI model for *ex vivo* organs, *InsightX*, which aimed to improve the assessment of organ suitability, donor-recipient matching, and post-transplant outcomes (48). The long short-term memory (LSTM) model was trained using 725 clinical cases and consisted of an ensemble of decision trees in a gradient boosting framework, making it more adaptable to handling missing values while ensuring high interpretability and performance efficiency. Three outcome classifications were used: time to extubation post-transplant <72 h, ≥72 h, and unsuitable for transplant. The model performed well in the prediction of post-transplant outcomes, achieving an area under the receiver operator curve (AUROC) of 79% ± 3% in the training dataset, with AUROCs of 75% ± 4% and 85 ± 3% in the independent test sets, with an AUROC of 90% ± 4% for the correct identification of unsuitable donor lungs (48). The team further developed the model with the addition of recipient data and used data intensive *in silico* models otherwise known as digital twins to run accurate simulations and allow for precise treatment selection. The additional data further improved the predictive accuracy of the model, resulting in no significant differences between the predicted and observed outcomes (*p* = 0.88). The results have been extremely promising, enabling more accurate identification of unsuitable organs and optimal donor-recipient matching, and improving clinical outcomes, and the team are currently working on further validation following the incorporation of radiographic data.

Although the 1-year survival of liver transplant recipients has improved over the last 30 years, long-term survival has remained largely unchanged (49, 50). Mortality beyond 1 year is predominantly due to issues with graft failure, cardiovascular events, and *de novo* and recurrent cancers (49, 50). This clinical conundrum motivated MB and her team to design *DynaComp* (Transplant Hepatology and Machine Learning Departments, University of Toronto, Toronto, ON, Canada), a personalized risk calculator for the long-term management of transplant recipients. Longitudinal data incorporating 267 clinical variables and 5 outcome measures were obtained from the SRT and used in the development of the training model, including overall survival, death by graft failure, death by infection, cardiac events, and cancer (51). Of the preliminary models, the *Transformer* model (Transplant Hepatology and Machine Learning Departments, University of Toronto, Toronto, ON, Canada) demonstrated the greatest predictive accuracy, with overall AUROCs for 1- and 5-year survival of 0.77 and 0.711, respectively. The AUROCs for survival compromised by cardiovascular disease, cancer, infection, and graft failure were between 0.80–0.81. Shapley additive explanations (SHAP) analysis was performed to determine the relative impact of each variable on the model output, revealing the highest weighted variables to be: time since transplantation, donor and recipient age, etiology of liver disease, and body parametric data including weight and BMI, in addition to standard laboratory results. Following analysis, the results are displayed in the form of a personalized dashboard, projecting estimations of 1- and 5-year survival, including a breakdown risk of death from graft failure, cardiovascular events, infections, and cancer. The model can serve as an invaluable prompt for physicians in clinic, guiding them to react to risks, adjust immunosuppression, and initiate primary prevention measures, with the added advantage of allowing them to assess the impact of the therapeutic modifications on subsequent visits, ultimately improving the preservation of graft health and overall survival.

### 2.2 Clinical deployment of machine learning tools: practical and ethical considerations in transplant

The afternoon session was opened by Professor Doug Simonetto from the Mayo Clinic, who spoke about the development of an electrocardiogram (EKG)-enabled machine learning model designed to improve prognostication in chronic liver disease. Advanced liver disease is associated with the evolution of cardiomyopathy and cirrhotic cardiomyopathy, which lead to specific EKG changes (52). Professor Simonetto and his group worked on a binary classification model with a convoluted neural network (CNN) for the identification of cirrhosis using 12-lead EKGs. The generated output was identified as an AI cirrhosis EKG (ACE) score, a continuous value ranging from 0 and 1, reflecting the estimated strength of the “cirrhosis” signal from each EKG (53). Data from 5,212 liver transplant recipients who had cirrhosis listed as an indication for transplantation and had a digitized EKG prior to transplant, combined with 20,728 age- and sex-matched controls, were used for the training, testing, and validation cohorts. Following analysis, an AUROC of 0.98 was achieved for the prediction of cirrhosis in the testing cohort, with a sensitivity of 84.9% and a specificity of 83.2%. When adjustments were made for one-to-one matching, including comorbidities, the performance of the model did not significantly change, with an AUROC of 0.893. Following on from this, the team looked to see whether there were any longitudinal changes in the score and determined that the ACE score continued to increase from 5 years before transplant, reaching a peak immediately prior to transplantation, after which it significantly declined. On this basis, the team proceeded to explore whether the score could have utility in prognostication and predict the future risk of decompensation and death. They obtained data from 500 patients with compensated cirrhosis predominantly due to metabolic liver disease (alcohol misuse and metabolic-dysfunction associated steatohepatitis), who had longitudinal follow-up that included EKGs. The model demonstrated an AUROC of 0.932 for the prediction of decompensation, with a sensitivity of 88.4% and specificity of 83.9 % (54). Following logistic regression, the team found that for every 0.1 increase in the ACE score, there was a 4.8× increase in the odds of clinical decompensation after adjusting for the MELD-Na score. The team then split the ACE scores into quartiles and assessed 1- and 5-year survival. Patients in the top quartile with ACE scores ranging from 0.75–1 had a >50% 1-year mortality, reaching almost 100% at 5 years, whereas those in the lowest quartile with ACE scores ranging from 0–0.25 had a <10% 5-year mortality. In the fully adjusted model, each 0.1 increase in the ACE score was associated with a 42% higher risk of liver-related death. The model performed well in predicting the risk of decompensation and death in individuals with compensated chronic liver disease.

Dr. Mike Brudno, chief data scientist at the University of Toronto, then provided an entertaining illustration of the challenges when training DL models, in particular the dangers of a “black box” classification model, using the team’s latest experience developing a model for the identification of pneumothoraxes from chest radiographs as an example. From initial iteration, the model had been devised using 7,000 images obtained from University Health Network (UHN) cases, of which 1,000 cases were positive for pneumothoraxes and 6,000 were negative. The model had initially been trained using a segmentation approach; however, this did not perform well, with a low Dice similarity coefficient score (coefficient of similarity between the “predicted” DL image and the “true” image ranging from 0 to 1) of 0.49, indicating a less than 50% chance of the model correctly identifying a pneumothorax, with a high standard deviation of 0.28 (55). Reviewing the initial images, the team noted that there was huge variability in the pneumothorax “negative” radiographs, with not enough data to apply the segmentation method; as a result, they used new data to update the model and switched to a classification-based method of analysis. In addition, the team modified the aim of the model to identify high-risk radiographs for the attention of radiologists, assisting with workflow prioritization. Testing the new iteration from 2,200 UHN scans, the AUROC of the model was 87.12%, with a sensitivity of 61.9% and specificity of 91.44%. Although the performance appeared acceptable, the team used gradient-weighted class activation mapping (Grad-CAM) to identify the x-ray regions the model focused on and deemed essential for the prediction of a pneumothorax in an image-based CNN model. They noted that the model was drawing incorrect inferences when using chest drains (the treatment for pneumothoraxes) or the presence of chest leads (usually associated with more acutely unwell patients) for its predictions. In essence, the model was erroneously determining causation from association. The team then adopted a multistep model; the first step involving the identification and removal of patients with chest drains and the second step involving the identification of pneumothoraxes. This really highlighted the importance of assessing explainability with clinically applied DL models.

The final talk of the day was given by Dr. Joseph Cafazzo, the executive director of biomedical engineering at the UHN, on the importance of human factors in the successful deployment of AI-based tools. Although there has been a plethora of products devised specifically for the healthcare industry, many have not passed the test of usability by failing to incorporate the consideration of human factors during the design phase. Human factors relate to the application of what is known about human capabilities and limitations from cognitive, behavioral, and environmental perspectives, in the design of the world we live in, to enable safe and productive lives. In particular, there tends to be a lack of empathy in the design of many healthcare tools. The challenge when it comes to the conception of these models is being mindful that it is not just a product but an experience that is being designed. One successful example includes the design of Medly (Centre for Digital Therapeutics, Toronto, ON, Canada), an application developed with Dr. Heather Ross that facilitates self-monitoring for patients with heart failure. Since its launch in 2016, the application has empowered patients and their families to take on a more active role in their health, improved quality of life, clinical outcomes, and healthcare-associated costs with a 50% reduction in the number of hospitalizations due to heart failure, and has demonstrated continued good uptake and adherence from the older patient population (56, 57). This demonstrates the potential impact of deploying practically useful AI tools on saving lives and reducing healthcare system costs.

## 3 Discussion

AI is a rapidly evolving area in healthcare. With the capacity to form complex non-linear analyses from large datasets and identify novel relationships, DL offers the potential to dramatically advance our diagnostic, patient monitoring, and prognostication techniques, thereby improving patient care. However, significant care is required in the development of these models, ensuring large and diverse derivation cohorts to reduce bias and guarantee generalizability, with appropriate explainability to confirm that the conclusions drawn by the model are coherent from a logical pathophysiologic perspective. In addition, regulatory bodies, including the FDA and Health Canada, need to devise specific policies for the appropriate governance of AI-based applications. Finally, the doctor-patient relationship has the potential to be enhanced when physicians can delegate clerical tasks to AI, leaving them free to focus on patient interactions.

## Data Availability

The original contributions presented in the study are included in the article/Supplementary Material, Proceedings of the 2024 Transplant AI Symposium further inquiries can be directed to the corresponding author.
